# Does a human rights-based approach to harm reduction support commercialized harm reduction? Brief research

**DOI:** 10.3389/fpubh.2022.1001036

**Published:** 2022-10-26

**Authors:** Neil Sircar, Mary E. Fleming, Stella A. Bialous

**Affiliations:** ^1^Public Health Law Center, Mitchell Hamline School of Law, Saint Paul, MN, United States; ^2^University of Minnesota, Minneapolis, MN, United States; ^3^Department of Social and Behavioral Sciences, UC San Francisco, San Francisco, CA, United States

**Keywords:** tobacco and tobacco product, human rights, harm reduction, public health, human rights-based approach (HRBA)

## Abstract

In recent years, the tobacco industry has been pushing a narrative that their newer lines of products—including electronic nicotine delivery devices—are offered in part to meet a social responsibility of providing potentially reduced-harm choices to their consumers. While some of the newer tobacco products might potentially be less harmful than combustible tobacco products, there is also significant deviation from the very concept of harm reduction when it is used for such a conspicuously commercialized purpose. The framing of commercialized tobacco harm reduction as a mere consumer preference by the industry is not clearly consistent with the core principles of harm reduction, let alone the human right to health and the highest attainable level of health. A human rights-based approach (HRBA) to harm reduction is a set of principles that frame an effort to respect and promote human rights, including the right to health. Whether the HRBA supports commercialized harm reduction requires study. We review industry materials from 2017 to 2022 to identify themes in the harm reduction narrative of the tobacco industry and analyse those themes using an HRBA to the tobacco harm reduction framework. Using this analysis, the industry's continued marketing of combustible products alongside their “potentially less harmful” products, and preference that their non-combustible products be regulated less strictly than cigarettes and cigars, adulterates the public health principles of harm reduction and undermines the right to health. We conclude that the tobacco industry's commercialized tobacco harm reduction is incompatible with a human rights-based approach to tobacco harm reduction.

## Introduction

In recent years, the tobacco industry has been pushing a narrative that its newer lines of products—including electronic smoking devices (ESD) and more broadly electronic nicotine device systems (ENDS)—are offered in part to meet a social responsibility of providing potentially reduced-harm choices to its consumers (1). The tobacco industry claims that its non-combustible products, such as electronic cigarettes, heated tobacco devices, snus, pouches, and chews, can help people who use cigarettes switch to a potentially less harmful alternative, thereby reducing the risks for harm from tobacco products (2). While some of these newer tobacco products are potentially less harmful than cigarettes, there is also a significant deviation of the very concept of harm reduction when it is used principally for a commercialized purpose (3). The tobacco industry itself has made clear that whatever benefit its “next-generation” products may provide, it coexists with the continued profit-making from manufacture, distribution, and sale of combustible tobacco products to consumers, with minimal if any marketing restrictions including on who may purchase these allegedly less harmful goods (e.g., non-smokers and first-time users) (4, 5). The tobacco industry wants to continue to sell harmful products directly to consumers while enjoying the social and political capital that may accrue from *also* selling potentially less—but still—harmful products directly to those same, and new, consumers.

### Harm reduction and commercialized harm reduction

Harm reduction principles emerged in the substance abuse and HIV/AIDS crises of the 1980's as a comprehensive and multi-faceted methodology towards supporting persons who engaged in risky behaviors (6). There is no single definition for harm reduction, although the principles that underscore harm reduction tend to be generalized and include commitments to human rights and social justice (5). Harm reduction absolutely includes a refrain from requiring abstinence as a precondition for receiving support or treatment. In addition, harm reduction encompasses a continuum of evidence-based social, physical, and mental healthcare that encourages positive individual change. It further includes respecting the rights of the persons who engage in the activity and delivering upon those rights through a strategy developed by and with those same persons.

Harm reduction has never meant the mere commercial or open-market offering of another addictive and harmful substance, in tandem with the original addictive and harmful substance, and in the absence of any social or medical support and infrastructure, and without cessation as an end-goal. Put more simply, there was never an offer of a less harmful heroin as a public health strategy to reduce the harm of using heroin—while offering both to the consumer at any given store. The strategy was to offer prescription methadone tied to a tightly regulated and overseen continuum of care, which encouraged cessation, one which included behavioral, medical, and social support along with naloxone and clean needles. Moreover, the principled harm reduction strategy did neither encourage or benefit from continued heroin use nor did it market opioids like heroin and methadone to the wider public like general goods anyone may choose from.

By contrast, the tobacco industry's take on harm reduction is to offer both combustible tobacco products and “potentially less harmful” non-combustible products to everyone, current smokers and not, and put the burden on the consumer to choose which to pay for, with little if any encouragement or support for cessation. The industry frames this approach to harm reduction as “a well-established public health concept that seeks pragmatic ways to minimize the impact of an inherently risky activity without stopping it entirely” (7). Variations in this sentence proliferate across the websites and publicly available documents for the major companies, often accompanying appeals to consumer choice and the benefits of “switching” from one marketed product to another, and silence on whether a consumer could, or should, choose not to consume tobacco products at all as an end-goal.

This is a key distinction between the public health principles of harm reduction and the commercialized model of harm reduction the tobacco industry proffers (1, 3). There are products—nicotine replacement therapy drugs (NRTs)—which more clearly fall into the category of harm reduction, but the industry does not wish its “next-generation” products to be considered drugs and regulated accordingly. While there is a basis to say that with behavioral and other support, these “next-generation” products can improve smoking cessation at least as well as NRTs (8), the absence of that support does not clearly demonstrate that smokers will end their tobacco product use (9) or break their nicotine dependence (10). In other words, harm reduction is not merely having a product that is *potentially* less harmful than another product and *can* be used to reduce tobacco product use or end nicotine dependence. Even the industry's own description of harm reduction is that it is a way toward an objective; it is not, by itself, the endpoint that can be neatly achieved through telling consumers to choose their experience.

Peeters and Gilmore (1) were early to note the “opportunistic” language that the industry employs in its framing that belies any genuine commitment to harm reduction. The authors of that study note that the industry's shift to favoring harm reduction framings was partly in response to increased regulatory scrutiny and public disdain, while at the same time, the industry voiced its desire to generate new product sales without “cannibalizing” existing tobacco product profits (1). Tan et al. (11) similarly observed that the tobacco industry has been angling ‘harm reduction' to include the continued selling of combustible tobacco products alongside the marketing and sale of non-combustible tobacco products, as part of the industry's strategy to influence discourse and policy (11). Indeed, the industry's harm reduction campaign itself arguably began after successfully influencing a 2001 report from the then-named Institute of Medicine (now National Academies of Sciences, Engineering, and Medicine) that stated, among other things, that public health interventions aimed at reducing the harm of continued smoking were appropriate considerations—thereby opening the door for the industry's commercialized harm reduction strategy (1, 12).

### The right to health and harm reduction

The tobacco industry has been increasingly comfortable using human rights language to promote itself, although it has not yet grappled with the right to health (13). The right to health is established in principle with the Universal Declaration of Human Rights, Article 25, and as a matter of international human rights law through a series of treaties, including Article 12 of the International Covenant on Economic, Social, and Cultural Rights (14). The right to health provides that each individual is entitled to the highest attainable level of physical and mental health. Effectuating the right requires the state—whether local, subnational, or national—to progressively and proactively protect, respect, promote, and fulfill human rights. In policy and practice, this means the state must actively work to create the conditions for a healthy life, including access to health services and freedom to consent. These requirements are further embedded in global tobacco control, including the WHO Framework Convention on Tobacco Control (15, 16) and the UN Sustainable Development Goals (17–19). Indeed, the Sustainable Development Goals (20), which were envisioned to support the international human rights framework including the right to health (17, 21), have as Goal 3.a the objective of strengthening the WHO Framework Convention on Tobacco Control (FCTC) and increasing its implementation (19), The FCTC includes Article 5.3 on eliminating tobacco industry interference in public health policymaking—which could include industry subversions around harm reduction (22, 23).

Harm reduction's relationship to the right to health is axiomatic. Everyone, whatever their activity or behavior, shares the same entitlement to the highest attainable level of health; harm reduction practices are means by which states can create the environmental, social, cultural, and legal conditions to enable all persons their enjoyment of that highest attainable level of health (24). Harm reduction however is not endorsement of the activity and does not pretend that a standard below the highest attainable level of health is sufficient so far as state actors go. This is where the industry and some advocates for commercialized tobacco harm reduction go awry in their analysis: the right to health does not include nuances for industry preferences to sell its products to consumers in the least regulated way, even where one harmful product is *potentially*—an important qualifier—less harmful relative to another harmful product (25–27).

At the same time, the obligations to fulfill the right to health are primarily on the state, who may act on this obligation through developing and enforcing regulations on the tobacco industry including limitations on what products it may lawfully market (27). Businesses have a fiduciary duty to their shareholders to produce value, which for the tobacco industry means the continued manufacture and sale of harmful and addictive products. Even so, businesses are increasingly expected to act socially responsible (28), even to the promotion of human rights, but this expectation does not supplant the duty on states to protect, respect, promote, and fulfill human rights.

### A human rights-based approach to tobacco harm reduction

Human rights-based approaches (HRBAs) are conceptual frameworks that can guide programs, projects, and policy implementation in accordance with human rights principles and human rights law (29). An HRBA to tobacco harm reduction could follow the general model for rights-based approaches to health (15, 30). The framework begins with the recognition of the right to health and the inescapable hazard that any tobacco product is to health. Whether the tobacco is burned, heated, chewed, or used to derive nicotine for e-liquid vaporization, that consumption is inherently unsafe and cannot be made safe; all tobacco products, new and old, contain harmful substances and are hazardous (27). There is no human right to purchase, sell, or otherwise use tobacco products—whatever may be said of the potential reduced risks that ENDS may provide to, and only to, current smokers (27, 31, 32). And, should there be such potential benefit for ENDS to smokers, an HRBA to tobacco harm reduction would see them regulated more like NRTs than mere commercial commodities (27).

There are two core elements with respect to an HRBA to health: (1) progressive realization using maximum available resources and (2) non-retrogression (29). An HRBA to tobacco harm reduction cannot regards as an endpoint any policy or practice that does not fulfill the right to the *highest* attainable level of health. An HRBA to any harm reduction begins and ends with the people engaging in and impacted by the harmful activity and their fundamental human rights and must meet the principles of progressive realization and non-retrogression in an accountable, equitable, and participatory manner.

The industry considers its harm reduction strategy to be supportive of health, as well as supportive of fundamental human rights, while also serving as a lucrative investment and market opportunity (33–35). We are uncertain if commercialized harm reduction can be considered a rights-based approach, particularly where it perpetuates health harms. In this article, we describe how the industry is using its commercialized harm reduction narrative and analyse commercialized harm reduction through a rights-based approach to a health framework as applied to tobacco harm reduction.

## Methodology

We searched the public documents and Internet webpages of the five biggest multinational tobacco industry companies: British American Tobacco (BAT), Philip Morris International (PMI), Imperial Brands (Imperial), Altria Group (Altria), and Japan Tobacco International (JTI). Our data and document collection were limited to those materials, in English, provided by the companies on their main corporate websites. Our search parameters were to identify content that related to harm reduction, generated by the companies themselves, from 1 Jan 2017 to 30 June 2022. Our key search terms were “harm reduction,” “reduced harm,” “less harmful,” and variations thereon. Our search period began on 1 May 2022 and concluded on 1 July 2022.

We read all pages, identified main themes, and highlighted how these themes were associated with harm reduction and human rights. From our collective experience, we know that the tobacco industry has discussed its commercialized harm reduction business in materials that also discuss sustainability, like environmental sustainability and achieving the UN Sustainable Development Goals. For this reason, we also reviewed pages and materials that primarily focus on sustainability. We exclude materials that are ancillary or unrelated to our rights-based analysis for tobacco harm reduction (e.g., financial reports).

## Results

We identified 297 documents and webpages across the five companies that mention either harm reduction (and its analogs, such as “reduced harm”) or sustainability (Table 1). As indicated in the middle column of Table 1, there is notable variation between the companies in its engagement in either of these themes, with Altria being the most prolific in its materials and JTI the least. The right-most column of the Table 1 reflects our vetting for materials that specifically include mentions for harm reduction. We found that about 81% (241) of the total number of materials initially collected included mentions for harm reduction. Here, we describe the key themes we identified from reviewing the industry's harm reduction content and quote exemplars of these themes from industry materials. Supplementary Table A provides further data on the materials we collected and how we assessed them.

**Table 1:** Number of documents and webpages discussing harm reduction.

**Company**	**Total number of documents/** **webpages that mention either “harm reduction” or “sustainability”**	**Vetted number of documents/** **webpages that discuss harm reduction**
British American Tobacco	63	48
Philip Morris International	72	66
Imperial Brands	57	37
Japan Tobacco International	18	11
Altria	87	79
Total	297	241

### Tobacco harm reduction as a limited venture

The gist of the industry's approach to harm reduction is sustained consumption of a potentially less harmful product. As claimed by Imperial Brands: “harm reduction, a pragmatic public health approach that focuses on reducing the negative impacts of an activity rather than eliminating the behavior itself” (36). PMI offers a very similar quote: “This approach—aimed at eliminating or reducing as much as possible the negative effects rather than the activity itself—is the essence of harm reduction” (37). BAT is specific that, although they talk broadly about tobacco products and harm reduction, they are most concerned with harm reduction from the consumption of cigarettes; discussing the harm of its other products, even if less than cigarettes, is minimal (38–40). For example, BAT states that “combustible products pose serious health risks. The only way to avoid those risks is to not start–or to quit–smoking. That's why we are changing: creating new products backed by science, that provide adult smokers with less risky alternatives” (41). Variations of this line show up in other BAT and other company materials, although we appreciate that BAT's quote above from their 2021 ESG report includes two footnotes that significantly nuance, at best, the benefits of their product. Across companies, the primary target of its harm reduction efforts is limited to smoking cigarettes, with the enticement to switch to another tobacco product rather than reduce, let alone cease, tobacco product use.

### Tobacco harm reduction as a consumer choice

The tobacco companies echo one another in identifying consumer choices and consumer preferences as the primary driver behind tobacco use (42, 43). PMI, who has webpages committed to countering hate in society (while, buried within, framing itself and its products as the victim of hate and unwarranted distrust) (44), contends that adult choice must allow for both the continued sale of cigarettes and the allowance of sales for potentially less harmful products—and anything else represents a form of authoritarian control (45). With no mention of their own company's commitment to lies, misinformation, and obfuscation—and having never really been trusted in the first place—while insinuating that public health policymaking is a partisan backroom affair, PMI's Senior Vice President Gregoire Verdeaux writes that after COVID-19

“*…If we are to achieve progress, society needs to determine how personal choice and government intervention can coexist to the benefit of all. This must start with the reestablishment of trust and respect for truth. Facts matter. And so does transparency. In matters of health especially, decisions regarding rules and regulations must not be made behind closed doors and must be centered on science and objective truths, not political bias or efforts to curry favor with particular centers of influence”* *(**45**)*.

BAT similarly adopts narratives that consumers should decide how to best achieve their satisfying experiences from commercial tobacco product consumption and may prefer potentially less harmful products or may prefer combustible cigarettes—absolving the manufacturer itself of any role in that decision (7, 46, 47), BAT also connects their harm reduction strategy to human rights, briefly, if significantly, noting that its harm reduction approach is in part aimed at addressing health risks and human rights (33). JTI builds on this sentiment and positions the human right to choose as fundamental to their harm reduction and sustainability strategies (48).

We note consumers can only choose from what is available to them, and the industry chooses first to make all its products available for commercial sale—those that are harm-promoting, and those that are potentially less-harm-promoting.

### Tobacco harm reduction as a public health regulatory and commercial opportunity

Across the study period, multiple documents and webpages from the companies extol their commitments to producing less harmful products. Some companies go as far as to link their commercial pursuit to public health objectives explicitly (36, 49, 50). Altria expressly sees themselves as part of the solution to smoking, while also demonstrating the profit incentive for them and undermining criticism of the patent and latent harms of their non-cigarette products, stating that

“*The FDA [US Food and Drug Administration], the public health community and tobacco manufacturers all have a role to play in addressing misinformation that hinders progress on harm reduction. We believe it is our responsibility to help create the conditions for harm reduction to succeed – through education, awareness and advocacy – as we build a strong portfolio of smoke-free products that satisfy adult smokers' evolving interests and preferences”* *(**51**)*.

JTI took a similar stance in a document submitted in response to tobacco regulations in the United Kingdom, stating that “Only by smokers moving away from combustible tobacco products to these potentially less harmful alternatives can population harm reduction be achieved” (52). Harm reduction here is framed as the endpoint for the company's strategy, implying a new equilibrium for acceptably sustained harm. Even so, the companies note limitations on their own approach to harm reduction, such as with Altria's opposition to reducing nicotine levels in its products (53).

The industry is critical of public health efforts to regulate its commercial products as it claims its ESD and other “next-generation” products could (or would) be less harmful than its cigarettes, though not so far as to say it does not support any regulation. Altria, for instance, states that its products should be regulated, but differently than other tobacco products, so as to encourage adult smokers to switch from one of their products to another one of their products—like the popular JUUL products, which Altria partly owns (54–56). Altria uses this generic appeal to adult smokers and their choice-making to both encourage industry-favorable regulation and discourage industry-unfavorable ones, public health (or at least the public health argument for restrictive regulations, e.g., prohibitions on flavors) notwithstanding (57–59).

Although harm reduction is often discussed in these industry-favorable terms, we did not find substantial discussion about cessation, non-initiation, or even the role that the “next-generation” products may play in helping reduce smoking beyond providing consumers choices in any given tobacco company's more harmful and still harmful products.

### Tobacco harm reduction as advancing sustainability

Each tobacco company considers harm reduction to be a focus area of its sustainability efforts. Sustainability works as a catch-all term; it can mean addressing climate change and reducing tobacco waste, while also meaning economic and community development, all alongside the financial and return-on-investment sustainability important to any shareholder-held enterprise (34, 35, 47, 60–62). The companies also connect their investments in new product development, supply chain oversights, and labor practices to the UN Sustainable Development Goals and further paint themselves as eager partners in helping states achieve those targets (41, 63–65). Imperial, for example, states that consumer health is a priority for them and as part of their Environment, Social, and Governance strategy “is aligned with the relevant United Nations Sustainable Development Goals” like SDG3 on Health (63).

The industry materials utilize sustainability as a framing to accomplish multiple ends, with potentially less harmful tobacco products being part of that. For example, BAT states they are promoting sustainability “by providing adult consumers with a range of enjoyable products that carry less risk than continuing to smoke cigarettes,” in the same context of the company's efforts to reduce its packaging and embrace a “green economy” (66). Imperial and Altria both consider reducing harm from their tobacco products a part of their environmental, social, and governance sustainability goals, which themselves are tied to long term business interests (63, 67).

## Discussion

We identified several themes in how the industry has approached its commercialized harm reduction narratives, but none of these fully capture the principles of harm reduction as laid out in public health. The industry's written strategy seems to include the term consistently and frequently and to treat that alone as sufficient proof that what it is saying is evidence-based and true.

### A human rights-based approach to tobacco harm reduction, applied

The HRBA is a set of principles that inform policy and program development. It takes as a given that everyone is entitled to their highest attainable level of health and imposes as a duty on state (and in some instances, non-state) actors to fulfill that duty in an accountable, equitable, and participatory manner. Our framework for the HRBA to tobacco harm reduction applies the two core elements identified earlier for the HRBA: (1) progressive realization using maximum available resources and (2) non-retrogression (29). Underpinning the HRBA to tobacco harm reduction is the fulfillment of the highest attainable level of health for the individual; “potentially less harmful” may be relatively better but is undeniably not the highest attainable level of health. Furthermore, the operation of an HRBA to tobacco harm reduction must be accountable, equitable, and participatory between the public authorities and the public itself.

This creates the first of several challenges to the industry: it is not accountable to the public. As businesses, it is accountable to shareholders who expect returns on their investment in tobacco product manufacture and sale. This runs counter, fundamentally, to public health objectives and is itself possibly irreconcilable (3, 68). Equity is also going to be difficult for the industry, given that it continues to make and sell combustible products alongside its potentially less harmful non-combustible products while blaming its own consumers for choosing poorly. Even were the industry to cease the production of its combustible products, the presumed benefits—if any—from non-combustible “next-generation” products depend on consumer consumption of and knowledge about them—which are both impacted by self-interested industry influence and marketing.

As to the core elements, progressive realization using maximum available resources is the closest to align with the industry's preference for its harm reduction strategy—except that progressive realization still regards the end goal to be the highest attainable level of health. If there is a condition better than what the industry advocates for, what it advocates for is not going to meet this element's requirement. As is true for harm reduction principles, cessation and non-initiation are the ultimate health objective, and achieving that becomes the framework for how a rights-based program for harm reduction is structured, with the provision of resources like social and medical support to aid an individual toward that end. Where replacement is ever considered an objective, it is done so for *current* smokers alone and requires strict guardrails on access and availability (27).

As we noted, the industry does not discuss cessation outside of performative statements discouraging smoking (it does not discourage using its smokeless products, including ESD). The industry continues to make and sell combustible products and wants to consider it changing that they *also* make other products, too, because in the industry's narrative, it is the consumer's own choice (and fault) to continue smoking and using the products the companies choose to sell.

And, while it may say that the best thing to do is not start—the industry is a business, and its shareholders demand sustained growth. Those consumers will need to come from somewhere. Perversely, this business fact perforates the second element on non-retrogression: *any* tobacco product, whether cigarette or not, is harmful to use or even be around when used. Were states to regulate “next-generation” products as the industry wishes—as freely available commodities, unattached to cessation and support programs—those states may contribute to deteriorations of the right to health for both tobacco product users and non-users alike.

### Is there any role that “next-generation” products may have to support public health?

The tobacco industry's pursuit of commercialized harm reduction as a harm reduction strategy has some support from public health professionals (9, 69–71). From their perspective, whatever risks the consumption of these products have will ultimately be less than the known risks from cigarettes. An HRBA to tobacco harm reduction could utilize “next-generation” products, even if they are only potentially less harmful, but not in the manner the industry advocates. Rather, as required by the right to health and a rights-based approach to health (30), a well-regulated system where *current* smokers, alone, are provided access to these products in an accountable and supportive program aimed at cessation *could* meet the test (27). This might align with both what public health professionals beholden to the industry's harm reduction narrative say is the potential benefit of these potentially less harmful products—a benefit realized only by smokers—and who the industry says these same products are intended for.

We do not expect this model to meet shareholder expectations, and so we do not expect the industry to adopt it in its regulatory advocacy. Consequently, the tobacco industry's use of harm reduction can only be described as a bastardization of the harm reduction principles it claims to endorse, and their commercialized harm reduction strategy is incongruent with a human rights-based approach to tobacco harm reduction.

## Conclusion

A human rights-based approach to harm reduction does not see a commercialization of harm reduction as protecting, respecting, promoting, or fulfilling human rights. Continued use of, and initiation with, a still harmful and highly addictive tobacco product is not, and can never be, the highest attainable level of health.

If the tobacco industry wants to be sincere in its support for harm reduction, it can start by globally ending its production and manufacture of cigarettes and cigars now and not on a yet to be determined future date. If it further wants credibility, rather than consider itself as an unjustly hated pariah, it might end all marketing of its products since—as it says—nobody should start using them, and so there is no need to advertise and promote them. It could further commit to making its products only available (and tightly regulated) as therapeutic aids, in conjunction with behavioral and medical support to promote reduced use and ultimately end nicotine dependence. We would approve if the industry does, but will not be surprised if it does not.

## Limitation

The study's analysis is limited to the self-reported documents and content therein provided by 5 tobacco companies, which were available on their websites at the time of collection.

## Data availability statement

The original contributions presented in the study are included in the article/Supplementary material, further inquiries can be directed to the corresponding author.

## Author contributions

NS and SB conceived the initial project and study plan. MF conducted primary data collection, qualitative content analysis, created tables based upon the data collection, and content analysis. NS provided review and guidance in data collection as well as conducted secondary data collection and analysis with SB, and prepared the initial draft of the manuscript. MF and SB provided revisions to the manuscript. All authors contributed to the article and approved the submitted version.

## Conflict of interest

The authors declare that the research was conducted in the absence of any commercial or financial relationships that could be construed as a potential conflict of interest.

## Publisher's note

All claims expressed in this article are solely those of the authors and do not necessarily represent those of their affiliated organizations, or those of the publisher, the editors and the reviewers. Any product that may be evaluated in this article, or claim that may be made by its manufacturer, is not guaranteed or endorsed by the publisher.
