# Supplementary material for: Does a human rights-based approach to harm reduction support commercialized harm reduction? Brief research
Source: Front Public Health. 2022 Oct 26;10:1001036. doi: 10.3389/fpubh.2022.1001036 (PMC9645767; doi:10.3389/fpubh.2022.1001036)
Supplement: Supplementary file 1 [file Table_1.pdf]

Table A. Tobacco Industry Webpages and Public Materials Discussing Harm Reduction

Company	Document/ Webpage Title	Year	"Harm Reduction" Mentioned	Sample Language/Notes	"Sustainability" Mentioned	Sample Language/Notes
BAT	Human rights and modern slavery		0	N/A	2	"Protecting human rights is a clear priority in our Group Sustainability Agenda for delivering a positive social impact. Our Agenda also focuses on other important environmental, social and governance (ESG) topics that are interrelated to human rights, including climate change, farmer livelihoods, health and safety, people and culture, and business ethics."
BAT	Human Rights Report 2020	2020	0	N/A	14	"Our Sustainability Agenda is integral to our Group strategy and reflects our commitment to reducing the health impact of our business through providing adult consumers with a wide range of enjoyable and less risky products."
BAT	Modern Slavery Statement 2021	2021	0	N/A	15	"Our Annual Report 2020 provides details of our evolved Group strategy, with sustainability at its heart. This includes our clear corporate purpose to build A Better Tomorrow by reducing the health impact of our business through offering a greater choice of enjoyable and less risky products."

BAT	Our strategic approach to sustainability		0	N/A	12	"we are clear that reducing the health impact of our business is our principal focus area"
BAT	Reducing the health impact of our business		1	"We know scientific substantiation is essential for our New Category products to make tobacco harm reduction a reality." "We are committed, as our principal focus area, to reducing the health impact of our business through offering a greater choice of enjoyable and less risky products for our consumers."	1	"We are committed, as our principal focus area, to reducing the health impact of our business through offering a greater choice of enjoyable and less risky products for our consumers."
BAT	ESG		1	Harm Reduction targets are: £5 bn by 2025 in New Categories revenues and 50m by 2030 consumers of our non-combustible products	1	Reducing the health impact of our buisness / Harm Reduction is first focus area of Our Sustainability Agenda.
BAT	ESG Report 2021	2021	170	"'Tobacco harm reduction' is a public health strategy that aims to minimise the harm caused by combustible cigarettes by encouraging adult smokers, who would otherwise continue to smoke, to switch completely to scientifically-substantiated, reduced-risk alternatives."	240	Reducing the health impact of our buisness / Harm Reduction is first focus area of Our Sustainability Agenda.
BAT	ESG Report 2020	2020	238	"To build A Better Tomorrow™, we must reduce the health impact of our business. We can do this by investing in and innovating our New Category products, offering a greater choice of enjoyable, potentially less risky products."	111	Reducing the health impact of our business / Harm Reduction is first focus area of Our Sustainability Agenda.

BAT	ESG Report 2019	2019	43	"Our strategy is focused on making available a broad portfolio of alternative new category products that satisfy consumers by providing pleasure, reducing risk and offering an increasing choice."	54	Reducing the health impact of our buisness / Harm Reduction is first focus area of Our Sustainability Agenda.
BAT	Sustainability Report 2019	2019	13	"Cigarettes carry serious health risks, but many adults continue to smoke, so we've long been committed to tobacco harm reduction.<br>"	107	"As our principal ESG focus area, we are committed to reducing the health impact of our business through offering a greater choice of enjoyable and less risky products for our consumers."
BAT	Sustainability Report 2018	2018	61	"We are committed to working to reduce the<br>public health impact of smoking, through<br>offering adult consumers a range of potentially<br>reduced-risk products (PRRPs)."	117	Harm reduction is one of the three pillars of Our Sustainability Agenda.
BAT	Sustainability Report 2017	2017	57	"The key issue for our business is the real and serious health risks of smoking. So harm reduction is about providing adults, who continue to smoke, with a choice of less risky sources of nicotine."	97	Harm reduction is one of the three pillars of Our Sustainability Agenda.
BAT	Stakeholder engagement		0	N/A	8	"Consumer information and campaigns, such as on the health risks of our products and youth access prevention" is part of sustainability.
BAT	A sustainable approach		0	N/A	14	Harm reduction is the "principal focus area" of BAT's ESG focus areas.

BAT	Harm reduction		4	"Tobacco harm reduction is a public health strategy that is about minimising the negative health impact of smoking."	0	N/A
BAT	Materiality		3	"Given the serious public health impacts of smoking, harm reduction is our principal focus area."	7	Harm reduction is the "principal focus area" of BAT's ESG focus areas.
BAT	How we report		0	N/A	5	"Our purpose is to build A Better Tomorrow™ by reducing the health impact of our business through offering a greater choice of enjoyable and less risky products for our consumers."
BAT	World-class science		6	"For reduced-risk products (RRPs) to fully contribute to tobacco harm reduction, it's essential that their reduced-risk status, compared to smoking, is adequately demonstrated by robust science."	0	N/A
BAT	Reduced-risk products		0	no explicit mention of harm reduction, but discusses reduced risk; "It is widely acknowledged that most of the harm associated with conventional cigarettes is caused by the toxicants in the smoke produced by the burning of tobacco."	0	N/A
BAT	Tobacco heating products		0	no explicit mention of harm reduction, but discusses reduced risk; "For example, a study commissioned by the UK Department of Health in 2017 found that people using THPs were exposed to around 50–90% less of the 'harmful and	0	N/A

				potentially harmful' compounds compared with conventional cigarettes."		
BAT	Vapour products		3	"Our scientific vapour data has been published in more than 80 peer-reviewed manuscripts and adds to the wealth of evidence supporting the category's role in Tobacco Harm Reduction."	0	N/A
BAT	Modern oral products		0	no explicit mention of harm reduction, but discusses reduced risk; "Laboratory chemical studies for our modern oral products show they produce substantially lower levels of toxicants than cigarette smoke and even lower levels than snus — a traditional oral tobacco product which is already regarded as a reduced risk alternative to smoking."	0	N/A
BAT	Understanding the comparative risks of our products		3	"Tobacco harm reduction is a public health strategy to minimise the negative health impact of conventional cigarettes. It recognises the important role that alternative sources of nicotine with lower health risks offer to smokers who may not otherwise want or choose to give up."	0	N/A

BAT	Regulation		2	"Along with the industry, governments and the public health community have a key role to play in maximising the potential for reduced-risk products (RRPs) to contribute to harm reduction."	0	N/A
BAT	A Better Tomorrow Innovation Hub to launch in Italy	2021	1	"This product portfolio [of reduced risk products], underpinned by world-class science, demonstrates our commitment to tobacco harm reduction."	0	N/A
BAT	Assessing the reduced-risk potential of our products	2019	1	"All of our science to date seems to be pointing in the right direction and is aligned with our ambition to transform tobacco by offering consumers a portfolio of less harmful products."	0	N/A
BAT	Building A Better Tomorrow Responsibly	2021	2	"Talking about harm reduction and regulation, Kingsley said: 'Governments and regulators have a huge role to play and we really want to work together. We want whole of society, multi-stakeholder solutions.'"	1	N/A
BAT	The importance of effective tobacco harm reduction strategy	2021	6	"The availability of scientifically-substantiated, less risky products such as vapour products, tobacco heating products and modern oral products are crucial to effective tobacco harm reduction. Product regulations should recognise the role these alternatives can play in harm reduction by ensuring that high quality product standards are enforced, that	0	N/A

				consumers have access to information to make informed choices and, critically, that underage use is prevented.”		
BAT	2021 is a critical year for tobacco harm reduction	2021	17	push for countries to not ban vapor products in the name of harm reduction -- "It would be hard to imagine anything more damaging to global tobacco harm reduction efforts than further exclusion of these alternative products."	1	N/A
BAT	Tobacco harm reduction in focus	2018	8	"Tobacco harm reduction is a public health strategy that is about minimising the negative health impact of conventional cigarettes . . . ." -- "At BAT, our Transforming Tobacco ambition is underpinned by our commitment to tobacco harm reduction and seeking to reduce the public health impact of smoking."	0	N/A
BAT	Accelerating our progress towards a sustainable future	2021	0	N/A	14	Promoting sustainability "by providing adult consumers with a range of enjoyable products that carry less risk than continuing to smoke cigarettes."

BAT	Campaign highlights how BAT is building A Better Tomorrow	2021	0	N/A	2	"We are committed to our purpose of building A Better Tomorrow™ by reducing the health impact of our business through providing a range of enjoyable and less risky products."
BAT	Building A Better Tomorrow	2020	0	N/A	1	"Last month, we announced our evolved corporate strategy and purpose to build A Better Tomorrow, by reducing the health impact of our business through offering a greater choice of enjoyable and less risky products."
BAT	Celebrating 20 years in the DJSI	2021	0	N/A	3	"Building A Better Tomorrow by reducing the health impact of our business is core to BAT's future sustainability."
BAT	We're listed in DJSI for 19th consecutive year	2020	0	N/A	4	"We are building A Better Tomorrow by reducing the health impact of its global business, which entails: Committing to providing adult consumers with a wide range of enjoyable and less risky products."
BAT	BAT's 2018 Sustainability Report underlines Group's commitment to Transforming Tobacco	2019	1	"The Report outlines the Group's work and progress across its three strategic focus areas of harm reduction, sustainable agriculture and farmer livelihoods, and corporate behaviour."	5	"The Report outlines the Group's work and progress across its three strategic focus areas of harm reduction, sustainable agriculture and farmer livelihoods, and corporate behaviour."

BAT	British American Tobacco only tobacco company featured in prestigious Dow Jones Sustainability World Index	2018	1	"We are incredibly proud that our world-class sustainability agenda continues to be recognised by such a prestigious external organisation – a testament to, among other things, our commitment to upholding the highest standards of corporate conduct across the world; to our tobacco harm reduction activities; and to ensuring we market our whole range of cigarette and potentially reduced-risk products in a responsible manner to our adult consumers."	6	"We are incredibly proud that our world-class sustainability agenda continues to be recognised by such a prestigious external organisation – a testament to, among other things, our commitment to upholding the highest standards of corporate conduct across the world; to our tobacco harm reduction activities; and to ensuring we market our whole range of cigarette and potentially reduced-risk products in a responsible manner to our adult consumers."
BAT	British American Tobacco underlines commitment to transforming tobacco in latest Group reports	2018	2	merely mentioned	5	N/A
BAT	British American Tobacco launches 2018 Harm Reduction Focus Report	2018	8	"the company is maximising the potential of its products to contribute to harm reduction by continuously investing in innovation, building reliable evidence backed by robust science, and driving responsible growth of the industry through collaborative efforts"	0	N/A
BAT	New Sustainability Report shows how BAT is responding to a changing world	2017	2	"The report outlines the work of the Group across three strategic focus areas of harm reduction, sustainable agriculture and	5	Harm reduction is a strategic focus area of sustainability.

				farmer livelihoods, and corporate behaviour."		
BAT	British American Tobacco is the only tobacco company featured in prestigious Dow Jones Sustainability World Index	2019	0	N/A	6	"Our position in the DJSI World Index is testament to our commitment to transforming our company by offering adult consumers an innovative range of potentially reduced-risk products."
BAT	BAT named in Dow Jones Sustainability Indices for 19th consecutive year	2020	0	N/A	10	"This announcement is a further demonstration of BAT's continued commitment to its purpose, to build A Better Tomorrow by reducing the health impact of its global business . . . ."
BAT	BAT launches sustainability agenda to deliver A Better Tomorrow	2020	1	"The Group's new Sustainability Agenda supports the company purpose to deliver A Better Tomorrow with a principal focus on harm reduction."	9	"The Group's new Sustainability Agenda supports the company purpose to deliver A Better Tomorrow with a principal focus on harm reduction."
BAT	BAT in Dow Jones Sustainability Indices for 20th consecutive year - and only tobacco company in World Index	2021	0	N/A	12	"Building A Better Tomorrow by reducing the health impact of our business is core to BAT's future sustainability. This is why we are accelerating our transformation away from combustible cigarettes and investing to provide our consumers with enjoyable, less risky products."
BAT	BAT launches ESG Week to amplify	2021	0	N/A	8	"BAT aims to reduce health impact of its business targeting

	sustainability progress					50m consumers of New Categories products by 2030"
BAT	British American Tobacco named as one of the most sustainable companies in the world	2020	0	N/A	9	"Our approach to sustainability is at the heart of our plans to build a long-term sustainable business and we have a clear purpose to provide consumers with a range of potentially less harmful products."
BAT	Harm reduction goals and performance summary		2	"We are committed to working to reduce the public health impact of smoking, through offering adult consumers a range of potentially reduced-risk products (PRRPs)."	0	N/A
BAT	Sustainability performance centre		3	"We are committed to working to reduce the public health impact of smoking, through offering adult consumers a range of potentially reduced-risk products (PRRPs)."	0	N/A
PMI	Sustainability at PMI		0	N/A	9	"The biggest and most pressing negative externality our strategy aims to address is the health impacts of cigarette smoking."
PMI	PMI’s Statement of Purpose	2022	1	"The Company is committed to scientifically substantiating the harm reduction potential of its smoke-free alternatives compared to cigarette smoking, including through rigorous preclinical and clinical assessments and sophisticated systems toxicology."	5	N/A

PMI	2022 Annual Meeting of Shareholders and Proxy Statement	2022	1	"The Company is committed to scientifically substantiating the harm reduction potential of its smoke-free alternatives compared to cigarette smoking, including through rigorous preclinical and clinical assessments and sophisticated systems toxicology."	30	"The final metric is the Company's Sustainability Index, which consists of . . . Product Sustainability" which includes " efforts to maximize the benefits of smoke-free products, purposefully phase out cigarettes, seek net positive impact in wellness and healthcare, and reduce post-consumer waste."
PMI	2017 Proxy Statement	2017	0	N/A	12	"We discuss with the Company's scientists and engineers their progress in designing multiple Reduced-Risk Product platforms, and in conducting rigorous scientific assessments of these platforms."
PMI	2020 Proxy Statement	2020	1	"The scientific evaluation to substantiate their harm reduction potential compared to cigarette smoking is based on rigorous pre-clinical and clinical assessments and sophisticated systems toxicology."	23	N/A
PMI	Our approach to sustainability	2022	0	N/A	13	The "product health impact" is part of the ESG framework.
PMI	Integrated Report 2021	2021	22	Population harm reduction results from a combination of less harmful products, responsible marketing, and access to smoke-free products.	244	The "product health impact" is part of the ESG framework.
PMI	PMI's ESG KPI Protocol	2022	0	N/A	119	The "product health impact" is part of the ESG framework.
PMI	PMI's 2025 Roadmap	2022	0	N/A	3	The "product health impact" is part of the ESG framework.

PMI	2021 Sustainability Materiality Report	2022	0	N/A	13	The "product health impact" is part of the ESG framework.
PMI	PMI’s 2021 Integrated Report introduces new sustainability strategy to accelerate its smoke-free and ESG ambitions	2022	0	N/A	1	The "product health impact" is part of the ESG framework.
PMI	Focusing on the priorities for PMI’s sustainability strategy	2022	0	N/A	10	The "product health impact" is part of the ESG framework.
PMI	TOP 10 TAKEAWAYS from PMI’s Integrated Report 2021: Transforming for good	2021	0	N/A	24	The "product health impact" is part of the ESG framework.
PMI	Inclusion and compromise needed to address society’s biggest issues	2022	2	"In the area of tobacco harm reduction, PMI has long advocated for a sensible approach that prioritizes the interests of current adult smokers and public health."	0	N/A
PMI	Reporting on sustainability		0	N/A	4	The "product health impact" is part of the ESG framework.
PMI	Impact of PMI’s expansion in wellness and healthcare on different forms of capital	2022	0	N/A	3	"[W]e will continue to leverage our life sciences capabilities to move from reducing harm toward doing good, with the goal of having a net positive impact on the world."

PMI	Association between the introduction of heated tobacco products and declines in cigarette smoking	2022	0	"HTPs have the potential to contribute to the reduction of smoking prevalence by transitioning large numbers of adult smokers away from cigarette use over time, thus complementing existing prevention and cessation programs by substituting the most harmful use of nicotine, smoking, with less harmful, non-smoked forms."	0	N/A
PMI	Japan: Beginning a Smoke-Free Era		1	"Innovation [in heated tobacco products] has paved the way to harm reduction for millions of adult smokers worldwide."	0	N/A
PMI	Nicotine science	2022	3	"Many scientific experts and public health organizations support the role of tobacco harm reduction as a complement to existing tobacco control measures. They also recognize the important role of nicotine in tobacco harm reduction to help smokers transition away from the use of cigarettes."	0	N/A
PMI	Growing scientific and regulatory consensus on tobacco harm reduction		14	"Today, a number of governments acknowledge that smoke-free products have a role to play in reducing the harm caused by smoking and have adopted a tobacco harm reduction approach."	0	N/A
PMI	Conduct R&D responsibly and transparently	2022	3	"To address concerns over the reproducibility of research results, we created a platform called INTERVALS specifically	0	N/A

				for tobacco harm reduction science."		
PMI	Evolving PMI's business to deliver value in the long-term	2022	4	"A complete and successful transformation requires us to move from a value proposition built around harm reduction toward one with a net-positive impact on society."	0	N/A
PMI	PMI leaders' reflections on 2021, and learnings for 2022		1	merely mentioned	4	N/A
PMI	What is tobacco harm reduction?	2022	9	"Advances in science, technology, and regulation have enabled society to reduce the adverse effects of continuing with potentially harmful behavior. This is known as harm reduction."	0	N/A
PMI	Philip Morris International backs U.K. government's efforts to accelerate country's smoke-free ambitions	2021	1	"The U.K. government's progressive approach to tobacco harm reduction has already resulted in one of the world's highest number of smokers who have switched to better alternatives like e-cigarettes, and one of Europe's lowest smoking rates."	0	N/A
PMI	Disarm hate. Embrace dialogue	2021	2	merely mentioned	0	N/A
PMI	Choice vs. control: A dichotomy that transcends the pandemic	2021	1	merely mentioned	0	N/A

PMI	Science deserves a seat at the table	2021	2	"Rather than celebrate what by any objective measure is a positive public health breakthrough, some special interest groups are prioritizing ideology, politics, and a desire for retribution over progress. Fixated on the fantasy of an entirely tobacco-free world, they have lost sight of the opportunity that exists today, refusing to accept the science behind these alternatives and rejecting harm reduction as a solution for better."	0	N/A
PMI	The public supports governments taking a new approach to reducing smoking rates	2021	1	merely mentioned	0	N/A
PMI	PMI’s 2020 Integrated Report shows progress toward accelerating the end of smoking	2021	1	merely mentioned	21	"The report highlights PMI’s most material sustainability topics, including the health impacts of our products—an aspect often not captured by external ESG assessments."

PMI	Access to smoke-free products	2021	1	"As of the end of 2020, we estimate that only 36 percent of adult smokers in the markets in which PMI commercialized IQOS were aware of the product’s features and benefits compared with smoking cigarettes. This lack of information arguably presents the single biggest hurdle to achieving tobacco harm reduction. It is also potentially the easiest to overcome if and when public health organizations decide to inform adult smokers—or allow them to be informed—about the benefits of smoke-free products."	0	N/A
PMI	Responsible marketing and sales practices	2021	1	"At PMI, we support regulation that supports tobacco harm reduction, as well as measures to discourage initiation, encourage cessation, and encourage smokers who do not intend to quit to switch to less harmful non-combusted alternatives to cigarettes."	0	N/A
PMI	Responsible and transparent R&D	2021	3	"Scientific integrity, rigor, and transparency are necessary to drive positive societal change. Evidence resulting from robust scientific assessments advances the debate on public health and tobacco harm reduction, to the benefit of adult smokers."	0	N/A
PMI	Driving change together	2021	1	merely mentioned	5	N/A

PMI	Product addictiveness	2021	2	"Many agree that tobacco harm reduction, as an addition to (and not a replacement of) existing tobacco control measures is the right route to take."	0	N/A
PMI	PMI aims to become a majority smoke-free business by 2025	2021	1	merely mentioned	0	N/A
PMI	Can innovative products like IQOS accelerate the decline of smoking?	2020	1	merely mentioned	0	N/A
PMI	Putting consumers first: Pivoting for the future	2021	1	merely mentioned	0	N/A
PMI	Harnessing positive tension can accelerate progress	2021	1	Regarding the tobacco harm reduction space: "untold opportunity is lost when some public health advocates and organizations refuse to objectively assess the evidence surrounding smoke-free products, ignoring the potential these better alternatives to cigarettes may represent for men and women who would otherwise continue to smoke."	0	N/A
PMI	2020 Concordia Annual Summit speech by André Calantzopoulos	2020	1	merely mentioned	0	N/A
PMI	An “exposé” without an exposé		1	merely mentioned	0	N/A

PMI	Community support	2019	1	"The [Foundation for a Smoke-Free World's] role, as set out in its corporate charter, includes funding research into the field of tobacco harm reduction, encouraging measures that reduce the harm caused by smoking, and assessing the effect of reduced cigarette consumption on the industry value chain."	4	N/A
PMI	Product health impacts	2019	3	"It is the responsibility of any manufacturer to assess and demonstrate that switching completely to a novel tobacco and nicotine product has the potential to reduce the risk of harm compared with continued smoking."	0	N/A
PMI	Our drive to unsmoke		1	"Some people abhor tobacco companies. We get that. But don't allow that antipathy to stand in the way of tobacco harm reduction. Don't hurt smokers in an effort to hurt us."	0	N/A
PMI	Robust science and rigorous testing		1	Risk reduction "covers the full spectrum of activities from initial product development to clinical trials to the monitoring of these products once they are on the market for a long-term assessment of their contribution to harm reduction."	0	N/A
PMI	Letter from the Chief Executive Officer	2019	2	"Harm reduction is an accepted approach in many areas of life: from reducing salt and sugar contents in food and drinks to	7	N/A

				lowering carbon emissions by switching to electric cars."		
PMI	Vaping: Origins, safety, ingredients, and regulations	2020	2	referencing The Global State of Tobacco Harm Reduction report	0	N/A
PMI	Prohibition at any cost?	2020	1	merely mentioned	0	N/A
PMI	PMI confirms deal to distribute KT&G's smoke-free products globally, to accelerate the achievement of a smoke-free world	2020	1	merely mentioned	0	N/A
PMI	Lessons learned from prohibition apply to efforts for a smoke-free future	2020	1	merely mentioned	0	N/A
PMI	Big and small businesses can solve big challenges together	2020	1	"Our new white paper—drawing on a December 2019 survey of 17,251 adults in 14 countries—shows a strong public appetite for governments to make faster progress toward tobacco harm reduction."	0	N/A
PMI	Unsmoke Your Mind: Pragmatic Answers to Tough Questions for a Smoke-Free Future	2020	3	"All tobacco- and nicotine-containing products must be regulated, but—to ensure optimal harm reduction—those regulations should reflect these products' relative levels of risk."	0	N/A

PMI	In Support of the Primacy of Science	2020	3	"In other instances, such as tobacco harm reduction, science may be eclipsed by politics or dogma in some quarters."	0	N/A
PMI	Unsmoke Your Mind		1	"Only when a large number of adult smokers switch to smoke-free alternatives can these products realize their full potential for population harm reduction."	0	N/A
PMI	On a new mission: to support veterans' causes and start vital conversations	2019	1	merely mentioned	0	N/A
PMI	In science we trust	2019	1	"Tobacco harm reduction and smoke-free products are for all adult smokers who would otherwise continue smoking."	0	N/A
PMI	Assessing tobacco harm reduction	2022	8	"This approach—aimed at eliminating or reducing as much as possible the negative effects rather than the activity itself—is the essence of harm reduction."	0	N/A
PMI	World Health Organization report on tobacco: Fact versus fiction	2019	1	Rebuttle of WHO's report on tobacco that found that heated tobacco products were "undermining successful tobacco control initiatives" by claiming that "Progressive tobacco control policy embraces the concept of harm reduction and aims at moving smokers away from cigarette smoke, being the primary cause of smoking related diseases."	0	N/A
PMI	Why cigarette sales are	2019	1	merely mentioned	0	N/A

	substantially declining in Japan					
PMI	We’re all part of the solution	2019	1	merely mentioned	0	N/A
PMI	Transparency in all we do		1	"The [Foundation for a Smoke-Free World's] role, as set out in its corporate charter, includes funding research into the field of tobacco harm reduction, encouraging measures that reduce the harm caused by smoking, and assessing the effect of reduced cigarette consumption on the industry value chain."	5	N/A
PMI	Harm reduction equation		2	"The harm reduction equation describes the criteria for making a significant impact on public health by converting the greatest number of existing adult smokers who would otherwise continue smoking to less-harmful alternatives."	0	N/A
PMI	A demand for less harmful alternatives to cigarettes	2019	0	no explicit mention of harm reduction, but a general discussion of less harmful alternatives	0	N/A
PMI	The facts about nicotine	2017	0	no explicit mention of harm reduction, but a general discussion of less harmful alternatives	0	N/A
PMI	Sustainability is the core of our company’s transformation	2022	0	N/A	2	"Seven years ago, we set out to create a new future for PMI—a future in which cigarettes would be obsolete, replaced by less harmful, science-based alternatives. In 2021, we kept our relentless focus on that

						ambition, dedicating 99 percent of our research and development and 73 percent of our commercial expenditure to smoke-free products."
PMI	Accelerating the pace of change: Committed to making cigarettes obsolete	2022	0	N/A	4	"Staying competitive in the cigarette category during this interim period provides the infrastructure needed to support our smoke-free growth, allowing us to create a future where cigarettes no longer exist."
Imperial	Driving transformational change through a challenger approach		0	"By listening carefully to smokers, respecting them as informed citizens, and then innovating at pace, we are improving our range of products with the potential to reduce harm."	0	N/A
Imperial	We start with the consumer		1	"Together, we are innovating at pace on new product improvements, packaging, brand experience and engagement, grounded in consumer insight. A clear example of this approach in action is in the roll-out of our Pulze system for heated tobacco, a fast developing category, which is potentially less harmful than traditional cigarettes."	0	N/A
Imperial	Our Strategy	2022	0	One pillar of their five-year strategy is about next generation products, but there is no explicit discussion of harm reduction.	0	N/A

Imperial	Healthier Futures		2	"Head of Harm Reduction and Engagement Grant O'Connell explains our commitment to harm reduction and our focus on offering a range of potentially less harmful products."	2	N/A
Imperial	ESG Strategy 2022	2022	10	"We are committed to strengthening our next generation products (NGP) and making a more meaningful contribution to harm reduction by offering adult smokers a range of potentially less harmful products."	6	N/A
Imperial	Annual Report and Accounts 2021	2021	16	"We have an exciting opportunity to make a meaningful contribution to harm reduction by building a targeted and sustainable business in [the NGP] market, offering potentially reduced risk products."	34	One pillar of their sustainability strategy is next generation products and reduced harm.
Imperial	Sustainability Performance Summary 2021	2021	2	One pillar of their sustainability strategy is next generation products and reduced harm.	23	One pillar of their sustainability strategy is next generation products and reduced harm.
Imperial	GRI and SASB Index	2021	2	specifically names reduced risk products "tobacco harm reduction products"	11	N/A
Imperial	The history of our challenger brands		1	"Another step towards harm reduction" was sale of tobacco-free oral nicotine products.	0	N/A
Imperial	Our ESG Strategy		2	"We are committed to making a meaningful contribution to harm reduction by offering adult smokers a better choice of potentially less harmful products."	2	Consumer health is an ESG priority.

Imperial	Our Focus Areas	2022	4	"[W]e address consumer health by offering adult smokers a range of products that have the potential of harm reduction at population level."	0	N/A
Imperial	Full Year Results Statement	2021	4	"Through our focused, consumer-led next generation products strategy, we are committed to making a meaningful contribution to harm reduction over time by offering adult smokers potentially reduced risk products."	1	N/A
Imperial	Pre-close trading update (October 2021)	2021	1	merely mentioned	0	N/A
Imperial	Half Year Results Statement	2021	5	" Our aim is to create a successful NGP business that meets consumer needs and, over time, can make a meaningful contribution to harm reduction. "	1	N/A
Imperial	Capital Markets Webcast	2021	2	merely mentioned	0	N/A
Imperial	Full Year Results Statement	2020	3	merely mentioned	1	N/A
Imperial	Annual Report and Accounts 2020	2020	14	"{H]arm reduction [is] a pragmatic public health approach that focuses on reducing the negative impacts of an activity rather than eliminating the behaviour itself."	26	Consumer health is an ESG priority.
Imperial	Investor Webinar: Sustainability Strategy and Farmer Welfare [Transcript]	2020	0	N/A	26	One pillar of their sustainability strategy is next generation products and reduced harm.

Imperial	AGM Update	2020	0	"We believe that NGP provides consumers with potentially less harmful alternatives to combustible tobacco and offers a significant growth opportunity over the medium term to complement our Tobacco business."	0	N/A
Imperial	CAGNY 2020 Conference [Transcript]	2020	1	The sustainability strategy pillar of reduced harm Next Generation Products " is about developing alternative products that are potentially less harmful to health."	3	One pillar of their sustainability strategy is next generation products and reduced harm.
Imperial	Stewardship Forum 2019	2019	0	N/A	2	One pillar of their sustainability strategy is next generation products and reduced harm.
Imperial	CAGNY 2019 Conference [Transcript]	2019	0	"For smokers: we're providing a satisfying, less harmful experience, endorsed by a trusted brand in blu and available where smokers want to buy it."	0	N/A
Imperial	Annual Report and Accounts 2019	2019	7	"A growing body of research shows flavours play a critical role in attracting and retaining adult smokers into the vaping category, directly contributing to tobacco harm reduction and declining smoking rates."	42	One pillar of their sustainability strategy is next generation products and reduced harm.
Imperial	Preliminary Results for the Year Ended 30 September 2019	2019	0	"Our focus on transitioning adult smokers to potentially less harmful alternatives to cigarettes is aligned to our purpose: to create something better for the world's smokers."	0	N/A

Imperial	Preliminary Results for the Year Ended 30 September 2018	2018	0	"We have an outstanding NGP portfolio and in creating something better for the world's smokers, we are encouraging them to transition to less harmful alternatives to cigarettes. "	0	N/A
Imperial	Annual Report and Accounts 2018	2018	1	"There is a tangible sense of excitement about how our Next Generation Products (NGP) operations are developing and the contribution our products are making to the global harm reduction agenda. Many smokers currently continue to choose to enjoy tobacco and we will keep providing them with the high quality products they expect. But our aim is to increasingly transition smokers to our NGP portfolio, which consists of products that are significantly less harmful than cigarettes."	36	One pillar of their sustainability strategy is next generation products and reduced harm.
Imperial	Building blu	2018	7	Claims that harm reduction is the largest driver behing people choosing to vape.	0	N/A
Imperial	Annual Report and Accounts 2017	2017	0	"During the year the FDA said it would begin public dialogue about lowering nicotine levels in cigarettes as a way of transitioning smokers to 'alternative and less harmful' sources of nicotine and we look forward to taking part in this dialogue."	7	N/A

Imperial	Market trials underway for heated tobacco products	2021	1	"Building a targeted and sustainable Next Generation Product (NGP) business is a key part of Imperial's new strategy and its commitment to make a meaningful contribution to harm reduction."	0	N/A
Imperial	Vapour pilot to build consumer insights	2021	1	merely mentioned	0	N/A
Imperial	Imperial Brands and Fontem Ventures committed to compliance with US vaping regulations	2018	1	merely mentioned	0	N/A
Imperial	Our Transformation To Unlock Value	2021	2	mentioned in the context of Next Generation Products	0	N/A
Imperial	Annual General Meeting 2021 [Presentation]	2021	2	mentioned in the context of Next Generation Products	0	N/A
Imperial	Imperial Brands and Fontem Ventures committed to compliance with US vaping regulations	2018	1	merely mentioned	0	N/A
Imperial	Interim Results 2021 [Presentation]	2021	6	mentioned in the context of Next Generation Products	3	One pillar of their sustainability strategy is next generation products and reduced harm.
Imperial	Annual General Meeting 2022 [Presentation]	2022	1	mentioned in the context of Next Generation Products	0	N/A

Imperial	Sustainability Performance Summary 2020	2020	1	mentioned in the context of Next Generation Products	23	One pillar of their sustainability strategy is next generation products and reduced harm.
JTI	Our Approach to Sustainability		0	N/A	17	One of the benchmarks for sustainability is health and well-being, which mentions reduced-risk products.
JTI	Our sustainability priorities		0	N/A	4	"Sustainability is an evolving journey for any company, something that is reflected in our constantly evolving RRP advances. In an era where the risks associated with cigarettes are well known, consumers are seeking out increasingly diverse smoking and vaping experiences."
JTI	Reduced-Risk Products – our vaping products		1	"Consumers all over the world vary in their needs, demands and desires, and where one individual might find combustible products meet those requirements, a growing number are looking to the benefits of Reduced-Risk Products (RRPs). This is usually due to the products' potential for harm reduction and/or an experience that is more tailored to their lifestyle or situation."	2	"RRPs are core to the sustainability of our business, as we need to meet ever-evolving, increasingly diverse consumer demands with the best and widest variety of smoking experiences."
JTI	Respecting and safeguarding human rights across our operations		0	"Offering our consumers broad choice, we are committed to developing and bringing to market products that reduce exposure to known harmful constituents in tobacco smoke and that we believe have the	8	N/A

				potential to lower the health risks of smoking."		
JTI	Integrated Report 2021	2021	0	"Needless to say, it is clear that RRP will be of significance in the industry; we expect that they will continue to gain increased attention and traction with consumers as these products have the potential to reduce the health risks associated with smoking by offering solutions that do not involve burning tobacco and produce no smoke and less odor. They thus significantly reduce not only the potentially harmful constituents in smoke generated from burning tobacco leaves, but also the inconvenience to non-smokers."	177	N/A
JTI	Integrated Report 2020	2020	0	N/A	186	"Expanding our product portfolio to satisfy increasingly diverse consumer needs and preferences" includes reduced-risk products.
JTI	Integrated Report 2019	2019	0	N/A	174	"We will be a total tobacco company offering consumers an even greater choice of products by focusing on quality, innovation, and reduced-risk potential."

JTI	Sustainability Report 2018	2018	6	"The success of any harm reduction intervention is dependent on two factors: its potential to reduce the risks associated with a particular activity and the number of people who adopt the intervention."	79	N/A
JTI	Sustainability Report 2017	2017	0	"Health risks associated with smoking are believed to be caused primarily by potentially harmful constituents found in smoke from burning of tobacco. We therefore believe that RRP, which do not involve combustion or smoke, have risk reduction potential, and offer real benefits to consumers, society, and our business."	78	N/A
JTI	JT Group Human Rights Report	2021	0	"In line with an increasing number of scientists,<br>we believe that non-combusting products have the potential to reduce the health risks associated with smoking."	48	N/A
JTI	Consultation on the Tobacco and Related Products Regulations 2016 and the Standardised Packaging of Tobacco Products Regulations 2015	2021	6	"Only by smokers moving away from combustible tobacco products to these potentially less harmful alternatives can population harm reduction be achieved."	0	N/A

Altria	Reduce the Harm of Tobacco Products		11	"The FDA, the public health community and tobacco manufacturers all have a role to play in addressing misinformation that hinders progress on harm reduction. We believe it is our responsibility to help create the conditions for harm reduction to succeed – through education, awareness and advocacy – as we build a strong portfolio of smoke-free products that satisfy adult smokers' evolving interests and preferences."	0	N/A
Altria	Remarks at 2021 Tobacco Science Research Conference	2021	21	"The data to date demonstrate that tobacco harm reduction will require products that meet adult smokers where they are – and that includes responsibly marketed products available with the<br>product attributes shown to encourage adult smoker transition, including flavors demonstrated<br>through science to appeal to adult smokers and nicotine levels that will meet adult consumer expectations."	0	N/A
Altria	Communicating Tobacco Health Effects		1	"For tobacco harm reduction to be successful, a marketplace of FDA-authorized reduced-risk products must be in place, with more information about the relative risks of those products being communicated to adult smokers."	0	N/A

Altria	2022 Annual Meeting of Shareholders [Presentation]	2022	3	Altria's work includes "advocating for tobacco harm reduction by encouraging the U.S. Food and Drug Administration (FDA) and other stakeholders to address the widely held misperceptions in society about nicotine."	1	N/A
Altria	2017 Annual Report	2017	2	merely mentioned	0	N/A
Altria	2018 Annual Report	2018	8	"We believe our investment in JUUL is a key part of the portfolio approach to tobacco harm reduction that we've been following for many years"	0	N/A
Altria	2019 Annual Report	2019	2	"With adult smoker demand for non-combustible alternatives, innovation and an appropriate regulatory framework, we believe we can make more progress on harm reduction in the next 10 years than we have in the past 50."	0	N/A
Altria	2020 Annual Report	2020	2	Altria's tagline and the title of the report: "From tobacco company, to tobacco harm reduction company"	1	N/A
Altria	2021 Annual Report	2021	1	Altria's tagline and the title of the report: "From tobacco company, to tobacco harm reduction company"	0	N/A
Altria	Notice of Annual Meeting of Shareholders and Proxy Statement	2022	14	"[A] comprehensive approach to tobacco harm reduction means that all adult smokers, regardless of background, demographics or financial means, have equitable	3	N/A

				opportunities to reduce the harm of smoking."		
Altria	Altria Makes $12.8 Billion Minority Investment in JUUL to Accelerate Harm Reduction and Drive Growth	2018	3	"We have long said that providing adult smokers with superior, satisfying products with the potential to reduce harm is the best way to achieve tobacco harm reduction. Through JUUL, we are making the biggest investment in our history toward that goal. We strongly believe that working with JUUL to accelerate its mission will have long-term benefits for adult smokers and our shareholders."	0	N/A
Altria	Reduce the Harm of Tobacco Products - 2021 Snapshot	2021	13	"We understand that harm reduction cannot succeed without responsible engagement with regulators, legislators, and the public health community. We are continuing our efforts to work with these and other stakeholders to create the conditions necessary for harm reduction to succeed in the U.S."	0	N/A
Altria	Reducing the Harm of Tobacco Products 2020 – Q2 2021 Performance Snapshot	2020	6	"2025 Goal: Create the conditions for tobacco harm reduction to succeed through external communications and engagement, science and advocacy."	0	N/A

Altria	ESG Reporting & Data		3	A 2025 ESG goal is "Create the conditions for tobacco harm reduction to succeed through external communications and engagement, science and advocacy."	1	A 2025 ESG goal is "Create the conditions for tobacco harm reduction to succeed through external communications and engagement, science and advocacy."
Altria	Remarks at Global Tobacco & Nicotine Forum	2019	16	"We recognize that the opportunity of harm reduction for adults is imperiled by the dramatic increase of underage use of vapor products."	0	N/A
Altria	Moving Beyond Smoking At-A-Glance		6	"Tobacco harm reduction can only be successful if smokers find less risky products satisfying. We continue to believe that no one single product platform is likely to satisfy adult smokers."	0	N/A
Altria	Adult Smokers 21+ Should Have Access to Better Choices		2	"For tobacco harm reduction to succeed, adult smokers who don't or can't quit should be encouraged to transition to FDA-authorized, reduced-harm products."	0	N/A
Altria	Our Voice & Actions		1	merely mentioned	0	N/A
Altria	About Altria At-A-Glance		1	"The future of our industry is about innovation, harm reduction and informed consumer choice."	0	N/A
Altria	Who We Are		1	"The future of the tobacco industry is about innovation, harm reduction and informed consumer choice."	0	N/A

Altria	Our Vision		1	"Today over 20 million U.S. adult smokers seek less harmful alternatives to cigarettes. This is a pivotal moment. With adult smoker demand for non-combustible alternatives, innovation and an appropriate regulatory framework, we have the opportunity to make more progress on harm reduction in the next 10 years than we have in the past 50 years."	0	N/A
Altria	2019 CAGNY Conference	2019	6	"JUUL is a key part of the portfolio approach to tobacco harm reduction that we've been pursuing for many years. We're focused on expanding choices for adult smokers with satisfying, non-combustible products that have the potential to reduce harm across three platforms: e-vapor, heat-not-burn, and smokeless products."	0	N/A
Altria	Our Product Platforrms		0	"We believe reducing harm for adult smokers can only be successful if these consumers find potentially reduced-risk products 'satisfying' – a term with wide-ranging definition based on the diversity of adult smokers' interests."	0	N/A
Altria	Science, Research & Development		2	"Science is at the core of Altria's approach to tobacco harm reduction. Our investment in research and technology supports our smoke-free product development and the science needed for product applications	0	N/A

				submitted to the Food and Drug Administration (FDA)."		
Altria	Public Policy Positions		2	"Altria's Vision sets the direction for all our legislative efforts. For us, Moving Beyond Smoking means advocating for a harm reduction future in which the industry is operating within science-based federal regulation, underage tobacco use continues to decline and adult smokers who don't quit are transitioning to FDA-authorized, reduced-harm products."	0	N/A
Altria	Comments to Docket No. FDA-2012-N-0143 (84 Fed. Reg. 38,032, August 5, 2019) – Harmful and Potentially Harmful Constituents in Tobacco Products; Established List; Proposed Additions	2019	7	"Especially when applied to noncombustible tobacco products, FDA should implement [harmful and potentially harmful constitutents] reporting requirements in a manner that supports tobacco harm reduction efforts."	0	N/A
Altria	2017 Corporate Responsibility Progress Report	2017	16	"After decades of our companies advocating for harm reduction, the FDA now acknowledges the continuum of risk for tobacco products and distinguishes between the harm associated	4	N/A

				with combustible versus noncombustible products."		
Altria	Investors Day 2017 Presentation	2017	11	The Harm Reduction Framework includes: constituent reduction, risk reduction to the individual, harm reduction to the population, the product, exposure & health risk, and the impact on the population.	0	N/A
Altria	Remarks by Billy Gifford, Altria's CEO to the Richmond Association for Business Economics	2022	14	"Harm reduction is a generally accepted approach to addressing public health issues. It's the belief that for those individuals who can't or won't stop a certain practice, the focus should be on transitioning them to a potentially less risky product. It is also a recognition that prohibitionist policies generally don't work."	3	Moving beyond smoking, to smoke-free products, is part of sustainability.
Altria	2018 Corporate Responsibility Progress Report	2018	39	"Providing adult smokers with superior, satisfying products with the potential to reduce harm is the best way to achieve tobacco harm reduction."	5	N/A
Altria	Investor Highlights 2018	2018	1	" We have long said that providing adult smokers with superior, satisfying products with the potential to reduce harm is the best way to achieve tobacco harm reduction. And that's what we intend to do."	1	N/A

Altria	Responsible Marketing		1	"'Responsibly leading' . . . is a key pillar of our underage tobacco prevention framework and imperative to tobacco harm reduction."	0	N/A
Altria	Altria Releases Its Inaugural Task Force on Climate-Related Financial Disclosures Report	2021	1	merely mentioned	1	Reducing Harm and Preventing Underage Use is a responsibility focus area.
Altria	Remarks by Billy Gifford, Altria's CEO at the Global Tobacco & Nicotine Forum	2021	14	"[T]he the issue of equity is fundamental to the harm reduction discussion. A comprehensive approach to harm reduction means that all adult smokers, regardless of background, demographics or financial means, have equitable opportunities to reduce the harms of smoking."	0	N/A
Altria	Altria Reports 2019 Fourth-Quarter and Full-Year Results; Provides 2020 Full-Year Earnings Guidance; Revises 2020 - 2022 Adjusted Diluted EPS Growth Objective; Revises Terms of JUUL Transaction	2020	1	"We enter 2020 with continued focus on harm reduction. We believe Altria's enhanced business platform best positions us to succeed under various future category scenarios."	0	N/A
Altria	Supporting Science-Based FDA Regulation		2	"For many years, we have understood that harm reduction cannot succeed without effective, comprehensive federal regulation of tobacco products."	0	N/A

Altria	Altria Holds Investor Day; Reaffirms 2017 Full-Year Earnings Guidance	2017	1	merely mentioned	0	N/A
Altria	Responsibility At-A-Glance		0	Reduce Harm of Tobacco Products is one of Altria's Responsibility Focus Areas.	0	N/A
Altria	Comments on the Citizen Petition of the National Tobacco Reform Initiative	2020	13	asking FDA to regulate HR products differently	0	N/A
Altria	Regulation of Flavors in Tobacco Products		3	need for multiple reduced risk products -- "To advance tobacco harm reduction, it is imperative to create a diverse marketplace of products to meet adult smokers' preferences. To advance tobacco harm reduction, it is imperative to create a diverse marketplace of products to meet adult smokers' preferences."	0	N/A
Altria	ALCS-Comments-to-Dkt-No-FDA-2017-N-6189-Tobacco-Product-Standard-for-Nicotine	2018	6	"We urge FDA to issue final rules and implement the pathways in a manner that advances tobacco harm reduction, clarifies key terms and requirements, and avoids unnecessary review delays."	0	N/A
Altria	NNN COMMENTS MASTER DRAFT for KP 5.13.17	2017	13	"Harm reduction based on the continuum of risk should be a cornerstone of FDA's regulatory policy to achieve the greatest and most sustainable benefit to the public health."	0	N/A

Altria	2018 CAGNY Remarks	2018	4	Heated tobacco "produces no ash and less odor, and represents a compelling harm reduction opportunity."	0	N/A
Altria	Altria's Response to Senate Letter	2019	3	"We firmly believe in the long-term harm reduction opportunity that e-vapor products offer adult smokers who are interested in migrating from cigarettes to non-combustible tobacco products."	0	N/A
Altria	Congress Enacted Tobacco 21 to Stop Teen Vaping, Idaho Should Do the Same	2022	5	"A harm reduction framework means not only reducing underage use, but also helping adult smokers who don't quit move to FDA-authorized reduced harm products."	0	N/A
Altria	Altria and Philip Morris International End Merger Discussions; Altria Provides Statement on JUUL Leadership Change; Tightens 2019 Full-year Earnings Guidance	2019	1	merely mentioned	0	N/A
Altria	More than Half of U.S. Population Now Governed by State Laws Raising Minimum Age of Purchase for All Tobacco Products to 21	2019	1	Raising the minimum legal sales age to 21 "will reduce underage access to [e-vapor] products. It will also pave the way for e-vapor products to realize their enormous harm reduction potential for millions of adult smokers 21 and older."	0	N/A
Altria	Annual Shareholder	2017	2	One of Altria's Responsibility Priorities is Tobacco Harm Reduction.	0	N/A

	Meeting 2017 [Presentation]					
Altria	2021 CAGNY Investor Presentation	2021	2	The Harm Reduction Framework includes: constituent reduction, risk reduction to the individual, harm reduction to the population, the product, exposure & health risk, and the impact on the population.	2	reducing harm of products is part of sustainability
Altria	2017 CAGNY Investor Presentation	2017	2	Altria's role in harm reduction is developing products that meet consumers' harm reduction product desires.	0	N/A
Altria	Nicotine Product Standard in Cigarettes		1	"We support the FDA’s stated goal to advance harm reduction through a process meant to 'encourage innovative, less harmful and satisfying non-combustible products for adults who need or want nicotine.'"	0	N/A
Altria	Remarks by Howard A. Willard III, Altria Group, Inc.’s (Altria) Chairman and Chief Executive Officer (CEO), at Altria’s 2019 Annual Meeting of Shareholders	2019	4	"JUUL is a key element of our portfolio approach to harm reduction. Today, more than 13 million<br>adults in the U.S. have chosen e-vapor products, demonstrating that the vision of switching adults to noncombustible alternatives can be realized."	1	N/A
Altria	2019 CAGNY Investor Presentation	2019	2	"Working with JUUL to Advance Harm Reduction" is a "Compelling Harm Reduction Opportunity."	0	N/A

Altria	ALCS-Submission-to-FDA-2017-N-6565-Comments-and-Attachments	2018	27	"Transitioning current adult smokers to noncombustible tobacco products is a cornerstone of the overall harm reduction framework announced by Commissioner Gottlieb. Flavors can help accomplish that objective because flavors play an important role in adult smokers' noncombustible tobacco product preference and use."	1	N/A
Altria	ALCS Comments to Docket No. FDA-2019-N-2854	2019	11	"The PMTA pathway is central to tobacco harm reduction."	0	N/A
Altria	Comments on Tobacco Products; Required Warnings for Cigarette Packages	2019	0	Altria argued that HeatSticks should be exempt from graphic warning rule because of their benefits to harm reduction.	0	N/A
Altria	Altria's Third-Quarter 2020 Earnings Conference Call	2020	1	"We continue to believe that e-vapor products, including JUUL, can play an important role in tobacco harm reduction."	0	N/A
Altria	Center for Tobacco Products – Campaigns Program	2021	1	merely mentioned	0	N/A
Altria	Remarks by Marty Barrington, Altria Group, Inc.'s (Altria) Chairman, Chief Executive Officer (CEO) and President, at Altria's 2018 Annual Meeting of Shareholders	2018	2	merely mentioned	0	N/A

Altria	Docket No. FDA-2021-D-0756 (86 Fed. Reg. 72,603, December 22, 2021) – Comment	2022	1	"To achieve our vision, we are committed to advancing tobacco harm reduction for all stakeholders and believe that adult smoker conversion requires a wide array of alternative, smoke-free products."	0	N/A
Altria	ALCS and JMC Request for Extension of Public Comment Period to Draft Guidance	2019	1	merely mentioned	0	N/A
Altria	Protect the Environment 2020 – Q2 2021 Performance Snapshot	2020	0	Reduce Harm of Tobacco Products is one of Altria's Responsibility Focus Areas.	1	N/A
Altria	ALCS Comments_FDA_2017_N_6107_Regulation_of_Premium_Cigars	2018	1	merely mentioned	0	N/A
Altria	Reducing Harm and Preventing Underage Use 2020-2021	2021	52	"We also have a responsibility to ensure that harm reduction is equitable. Equity is fundamental to the harm reduction discussion. A comprehensive approach to harm reduction means that all adult smokers, regardless of background, demographics or financial means, have equitable opportunities to reduce the harms of smoking."	2	N/A
Altria	Remarks by Billy Gifford, Altria Group, Inc.’s (Altria) Chief	2022	5	"For harm reduction to succeed, it must create an off-ramp from cigarettes for adult smokers."	3	N/A

	Executive Officer (CEO), at Altria's 2022 Annual Meeting of Shareholders					
Altria	Annual Shareholder Meeting 2022 Presentation	2022	3	Altria's work includes "advocating for tobacco harm reduction by encouraging the U.S. Food and Drug Administration (FDA) and other stakeholders to address the widely held misperceptions in society about nicotine."	1	N/A
Altria	2022 CAGNY Remarks	2022	9	"For harm reduction to succeed, we believe smoke-free products must meet consumer expectations and deliver: enjoyable sensory experiences and nicotine satisfaction; reduced health risks and clear, authorized information about the benefits of switching; and the ability to avoid social friction associated with cigarettes, such as smoke odor, ash and feelings of isolation."	0	N/A
Altria	Lobbying and Political Activity Transparency & Integrity - Annual Report 2021	2021	15	"For tobacco harm reduction to succeed, adult smokers who don't or can't quit should be encouraged to transition to FDA-authorized, reduced-harm products."	3	N/A
Altria	Remarks by Billy Gifford, Altria Group, Inc.'s (Altria) Chief Executive Officer	2021	5	"Each consumer's journey to a non-combustible product will be different. So we will use responsible, tailored marketing approaches, personalized	2	N/A

	(CEO), at Altria’s 2021 Annual Meeting of Shareholders			customer support and evolve our products to advance harm reduction."		
Altria	2021 CAGNY Remarks	2021	4	"We believe the tobacco consumer’s understanding of the relative risks of non-combustible products compared to cigarettes is critical to achieving harm reduction."	1	"In summary, we believe the long-term sustainability of our businesses depends on our ability to deliver comprehensive solutions to critical environmental, social and governance challenges impacting a broad range of stakeholders."
Altria	Engage and Lead Responsibly 2020-2021	2021	15	Reduce Harm of Tobacco Products, which includes creating the external conditions for tobacco harm reduction success, is one of Altria's Responsibility Focus Areas.	11	Reduce Harm of Tobacco Products, which includes creating the external conditions for tobacco harm reduction success, is one of Altria's Responsibility Focus Areas.
Altria	Engage & Lead Responsibly: 2021	2021	13	Reduce Harm of Tobacco Products, which includes creating the external conditions for tobacco harm reduction success, is one of Altria's Responsibility Focus Areas.	19	Reduce Harm of Tobacco Products, which includes creating the external conditions for tobacco harm reduction success, is one of Altria's Responsibility Focus Areas.
Altria	Altria Reports 2021 Second-Quarter and First-Half Results; Narrows 2021 Full-Year Earnings Guidance	2021	2	merely mentioned	0	N/A
Altria	Task Force on Climate-related Financial Disclosures Report: 2020-2021	2021	3	merely mentioned	4	N/A

Altria	2019 Corporate Responsibility Progress Report	2019	34	"We've built a diversified non-combustible business model to further our tobacco harm reduction goals."	20	Reducing the Harm of Tobacco Products is part of sustainability and responsibility focus areas.
Altria	Remarks by Billy Gifford, Altria Group, Inc.'s (Altria) Chief Executive Officer (CEO), at Altria's 2020 Annual Meeting of Shareholders	2020	4	"We know that the tobacco harm reduction opportunity for adults simply cannot be achieved if underage tobacco prevention is not a priority for all stakeholders."	1	N/A
Altria	Altria Reports 2020 Third-Quarter And Nine-Months Results; Tobacco Businesses Demonstrate Resilience; Narrows 2020 Full-Year Earnings Guidance	2020	1	"Altria continues to believe that e-vapor products, including JUUL, can play an important role in tobacco harm reduction."	0	N/A
Altria	Altria's Fourth-Quarter 2021 Earnings Conference Call	2022	2	Depicting the "Risk Cliff Between Combustible & Smoke-free Tobacco" as very steep.	0	N/A
Altria	Prevent Underage Use		1	Preventing underage use of tobacco products "preserve[s] the opportunity for adult tobacco harm reduction."	2	N/A
